# Prevalence and Pattern of Oral and Maxillofacial Pathology in Al-Qassim Region, Saudi Arabia

**DOI:** 10.1155/2024/6611349

**Published:** 2024-09-24

**Authors:** Faraj Alotaiby, Rahaf Alruhaimi, Norah Alzamil, Ezdyan Alsemanni, Areej Almutairi, Hala Elsaka

**Affiliations:** ^1^ Department of Oral and Maxillofacial Diagnostic Sciences College of Dentistry Qassim University, Qassim, Saudi Arabia; ^2^ College of Dentistry Qassim University, Qassim, Saudi Arabia; ^3^ Department of Histopathology Laboratory King Fahad Specialist Hospital, Buraidah, Saudi Arabia

**Keywords:** malignant neoplasm, mouth mucosa, oral health, pathology, prevalence, retrospective studies, squamous cell carcinoma

## Abstract

**Objective:** The purpose of this study is to determine the prevalence and features of oral and maxillofacial lesions found in the residents of Al-Qassim region, Saudi Arabia.

**Methods:** A retrospective study was conducted at King Fahad Specialist Hospital, Buraidah, Qassim, KSA. The data for all biopsied oral and maxillofacial lesions were retrieved from January 2014 until August 2022. All patients' data including age, gender, location of the lesion, and histopathologic diagnosis were reviewed and analyzed using IBM SPSS version 23 and Microsoft Excel.

**Results:** A total of 381 oral pathology biopsies for individuals aged 18 and above were included in a descriptive analysis. One hundred ninety five (51.18%) of patients were male, and 186 (48.82%) were female. The site most commonly biopsied was the oral mucosa (26%). The diagnosis was categorized according to the histopathological diagnosis into 13 categories including all pathological lesions in the oral and maxillofacial area. The frequently biopsied category was soft tissue pathological lesion category (26%), second to that is the odontogenic cyst category (22%), and third is the immunological-mediated lesion category (13%). The sub-diagnosis that was mostly observed was radicular cyst, lichen planus, and focal fibrous hyperplasia with the percentages of 13.6%, 10.8%, and 9.4%, respectively.

**Conclusion:** The findings provide important information about the oral and maxillofacial pathology in Al-Qassim region, Saudi Arabia. This study found that biopsied oral lesions were more prevalent in males and in patients in the fourth decade of life. The oral mucosa was the most biopsied site, and the majority of the biopsies were soft tissue pathological lesions and radicular cyst was the most frequent diagnosis. Knowledge of such demographic and clinical features of oral and maxillofacial pathology cases helps in prediction of disease incidence and subsequent proper patient care in the region.

## 1. Introduction

Oral health is essential to the quality of life of individuals. Oral lesions can disrupt daily activities by causing pain and discomfort during essential functions like mastication, swallowing, and speech. Additionally, other issues like unpleasant odors, dry mouth (xerostomia), and unusual sensations (dysesthesia), can also disrupt a person's daily activities [1]. Due to their widespread prevalence and incidence across regions globally, oral diseases are recognized as significant public health concerns [2]. The oral cavity can host a diverse range of pathological lesions, each with distinct origins and characteristics [3]. The oral and maxillofacial regions are susceptible to various irritants that can result in diverse injuries, consequently contributing to a broad spectrum of pathology [4].

There are many different types of diseases that affect the oral cavity, including benign, reactive, developmental, and malignant lesions. Many lesions and mucosal diseases can be difficult to diagnose. To enable accurate differential diagnosis and effective patient management, determining the prevalence of oral and maxillofacial disorders is of paramount importance. This would rise awareness of disease patterns within a population among oral healthcare professionals [5]. Oral mucosal lesions may be caused by an infection (bacterial, viral, or fungal), local injury or irritation (traumatic keratosis, irritational fibroma, burns), systemic disease (metabolic or immunological), or lifestyle habits such as using tobacco, areca nuts, betel nut, or alcohol. Baseline data about the scope of the disease are needed for the planning of national or regional oral health promotion as well as for the prevention and treatment of oral health issues [6].

The diagnosis of some oral lesions can be challenging [5]. For many lesions, especially malignant diseases, a biopsy is essential for obtaining a definitive diagnosis [7]. Definite diagnosis of oral and maxillofacial lesions is typically based on complete data including clinical, radiographic, and microscopic examination of the biopsied specimen [8]. It has been proven that clinical examination alone is insufficient for identifying such lesions, more advanced techniques are needed to detect lesions early and treatment which could reduce morbidity and mortality rates [9]. To identify the prevalence and characteristics of oral and maxillofacial disorders in a certain population, it is significant to evaluate the prevalence of oral and maxillofacial lesions in a specific geographic area [10]. Assembling of demographic data and features of various diseases originating from oral and maxillofacial region is essential to build the demographics that can aid in differential diagnosis, patient management, treatment plan, and allocation of oral health services and promotion programs [5, 8, 11]. In Saudi Arabia, nearly all articles have considerably concentrated on the prevalence of oral cancerous lesions [12–16]. Only few studies have addressed the prevalence of oral and maxillofacial pathology in general [4, 10, 17–19]. The aim of this study is to determine the incidence and features of oral and maxillofacial lesions found in people residing in Al-Qassim region, Saudi Arabia.

## 2. Material and Methods

This retrospective study was conducted at King Fahad Specialist Hospital, Buraidah, Qassim, KSA. The data for all biopsied oral and maxillofacial lesions were retrieved from January 2014 to August 2022. A total of 381 biopsy were taken from patients with maxillofacial lesions who had been referred to the maxillofacial pathology department at KFSH. The study included adult patients aged 18 and above with complete data forms. Pediatric and adolescent patients aged 0–17 years, as well as patients with incomplete reports and those requiring further investigation for definitive diagnosis, were excluded from the study. Ethical approval was obtained from the ethical committee in health cluster in Qassim (approval number: 607-23-645). The data were recorded for all biopsies, including information on age, gender, lesion location, and histopathological diagnosis. Statistical analyses were conducted using SPSS statistical software (version 23) and Microsoft Excel. The data were subjected to an ANOVA test at a 95% confidence level.

## 3. Results

**Figure 1:**
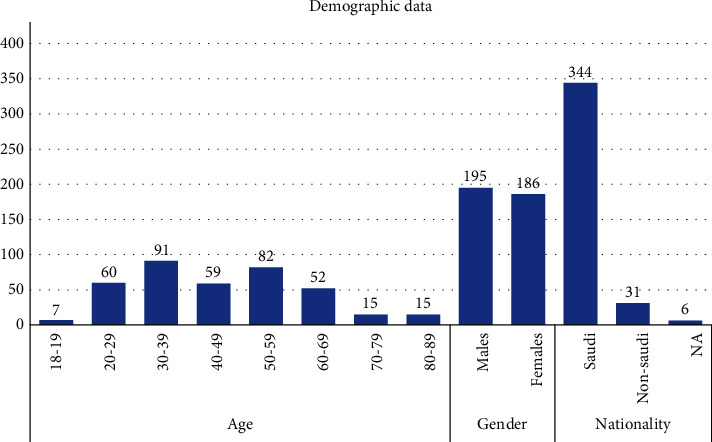
Demographic variables of the study sample.

A total of 381 oral pathology biopsies for individuals aged 18 and above were included in a descriptive analysis. 51.18% (*N* = 195) of patients were males and 48.82% (*N* = 186) were females. The age of the patients ranged from 18 to 88 years with a mean age of 47.6 years. Regarding nationality, the majority of individuals (*N* = 344, 90.29%) were Saudi, while 8.14% (*N* = 31) were non-Saudi. Information on nationality was missing in *N* = 6, 1.57% of cases (Figure 1).

The maxillofacial site most frequently affected by oral pathological lesions include:

• Oral mucosa NOS (*N* = 96, 26%)
• Mandible (*N* = 88, 23.2%)
• Maxilla (*N* = 64,17%)

**Figure 2:**
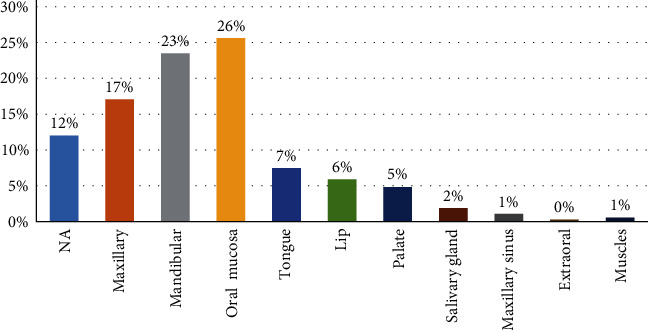
Distribution of lesions according to site.

The sites of (*N* = 45,12%) cases were unknown as shown in Figure 2. The maxillofacial lesions were classified into 13 categories, encompassing all pathological lesions within the oral and maxillofacial region. The most frequently biopsied categories include:

• Soft tissue pathological lesion category (*N* = 98, 26%)
• Odontogenic cyst category (*N* = 84, 22%)
• Immunological-mediated lesion category (*N* = 51, 13%)

**Figure 3:**
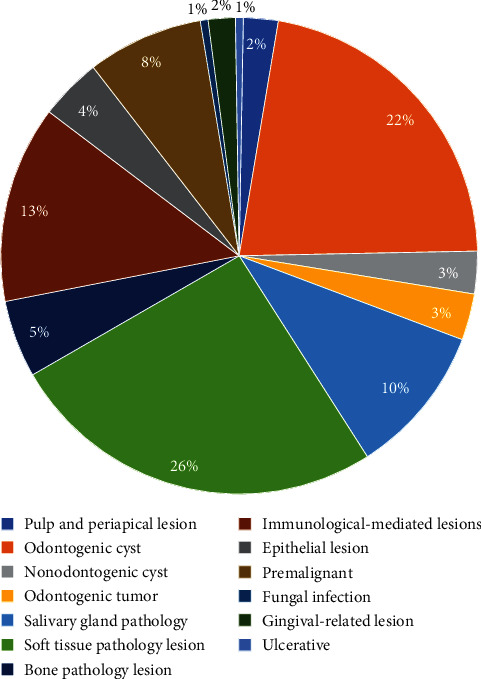
Distribution of lesions main diagnosis according to the categories.

The complete list of the categories is shown in Figure 3. The most common diagnosis of maxillofacial lesions include:

• Radicular cyst (*N* = 52, 13.6%)
• Lichen planus (*N* = 41, 10.8%)
• Focal fibrous hyperplasia (*N* = 36, 9.4%)
• Pyogenic granuloma (*N* = 31, 8.1%)

List of the diagnosis of maxillofacial lesions is presented in Table 1.

Oral mucosa NOS as a site of oral lesion accounts for *N* = 95, 25.0%. The most frequently observed lesions in the oral mucosa NOS were lichen planus, focal fibrous hyperplasia, and pyogenic granuloma, with the frequency of *N* = 31, 32.6%, *N* = 15, 15.8%, and *N* = 11, 11.6%, respectively. Table 1 shows that the mandible is the second in site *N* = 88, 23.2%. The most frequently seen lesions in the mandible were radicular cyst, dentigerous cyst, and pyogenic granuloma, with the frequency of *N* = 15, 17%, *N* = 11, 12.5%, and *N* = 8, 9.1%, respectively. The maxilla is the third in the most frequently affected sites *N* = 64, 16.8%. The most common lesion in the maxilla was radicular cyst, nasopalatine, periapical granuloma, and pyogenic granuloma with the frequency of *N* = 27, 42.2%, *N* = 5, 7.8%, *N* = 4, 6.3%, and *N* = 4, 6.3%, respectively. Other sites affected by maxillofacial lesions are tongue with the most prevalent lesions equally is squamous cell carcinoma and focal fibrous hyperplasia *N* = 5, 17.9%. Lip with the most common lesion equally is mucocele and pyogenic granuloma *N* = 4, 18.2%. Lastly, the palate with the most frequent lesion being focal fibrous hyperplasia *N* = 5, 27.8%. In the odontogenic cyst category, the most common diagnosis was inflammatory odontogenic cyst *N* = 55, 14.44%, followed by developmental odontogenic cyst *N* = 24, 6.30%, and last is miscellaneous NS odontogenic cyst *N* = 5, 1.31%. In the nonodontogenic cyst category, the most common diagnosis was nasopalatine cyst *N* = 7, 1.84%, followed by nonodontogenic cyst miscellaneous *N* = 4, 1.05%. In the salivary gland pathology category, salivary gland syndrome-related lesion such as Sjogren's syndrome and salivary gland idiopathic were the highest frequent diagnosis with *N* = 12, 3.15%. In the odontogenic tumor category, ameloblastoma was the highest diagnosed lesion *N* = 7, 1.84% followed by odontoma *N* = 2, 0.52%. In the soft tissue pathology category, reactive soft-tissue lesions were the most common diagnosis *N* = 73, 19.16%. In the bone pathology lesion category, the most frequent diagnosis is bone tumor/tumor-like lesions *N* = 10, 2.62% followed by bone infection/inflammation *N* = 9, 2.36%. Epithelial benign tumor-HPV-related lesions were the most common diagnosis in the epithelial lesion category *N* = 10, 2.62%. As for the premalignant/malignant category, epithelial malignant tumor was the most common lesion *N* = 24, 6.30%. Lastly, the gingival-related lesion category, the most common diagnosis is by equal the gingival infection/inflammation and gingival-related lesion-miscellaneous idiopathic NS *N* = 3, 0.79%. The complete list is shown in Table 1.

**Figure 4:**
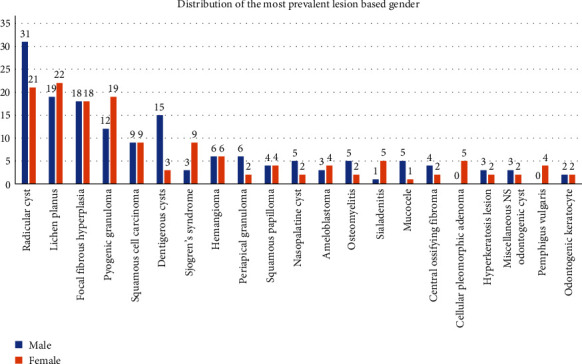
Distribution of lesions-based gender.

Based on the distribution of lesions by gender, lichen planus (*N* = 22) was the most common lesion in female patients, followed by radicular cyst and pyogenic granuloma. Whereas, radicular cyst (*N* = 31) was the most frequent lesion in males (Figure 4).

## 4. Discussion

The aim of the current study is to present the prevalence and clinical features of oral pathologic lesions in Al-Qassim region, Saudi Arabia during the period 2014–2022. To the best of our knowledge, this is the first study of such issue conducted in Al-Qassim. It is essential to document the prevalence and pattern of oral and maxillofacial pathology presented in a certain population for disease incidence prediction and proper oral healthcare. The main hospital that receives referral patients from the entire region of Al-Qassim province is King Fahad Specialist Hospital. It is the only hospital in Qassim that provides biopsy service during the last decade. In this study, the prevalence of oral and maxillofacial lesions was slightly higher in males (51.18%) when compared with females (48.82%), contrary to previous studies conducted in Saudi Arabia, where female predominance was present [4, 10]. In our study, oral lesions were most common in the fourth decade of life (23.88%), which was also observed in another study in Kuwait [20]. Approximately 90.29% of these lesions were registered in Saudi patients, with 8.14% in non-Saudi patients, and this low percentage of non-Saudi patients was also seen in a study carried out in southwestern of Saudi Arabia, where 6.1% of the patient were recorded as non-Saudi [4]. The present study shows that oral mucosa NOS is the most frequently biopsied site (26%) and this was seen in a study conducted in Kuwait as well where the majority of lesions were found in labial/buccal mucosa site and mouth NOS [20]. On the other hand, other studies in KSA and Pakistan reported that the tongue is the most common biopsied site [4, 21].

Interestingly, both of these studies also showed that more than third of the lesions were malignant. This might reflect the difference in the biopsy protocol in the biopsy service that was involved in their studies, in which only high-risk lesions were biopsied. In this study the most frequently biopsied category was soft tissue pathology lesion category (26%), that contains reactive lesions (19.16%) as being the most frequent subcategory present, which is a similar finding to previous studies carried out in both Nigeria and Iran, 23.1%, 34.6%, respectively [22, 23]. Then, odontogenic cyst category as the second most biopsied category (22%), which was also supported by a previous study in an Australian adult population, where odontogenic cyst category was 16.3% of all OMF lesions [24]. In regards to the diagnosis, radicular cyst was the most prevalent diagnosis reported in our study (13.6%), which was in agreement with a previous study conducted in Spain accounting for 16.7% of cases [25]. However, this finding was in contrast with Kelloway et al. [24], where the most common diagnosis was fibrous hyperplasia, followed by chronic periapical granuloma, and the third was radicular cyst, 15.2%, 9.6%, 9.5%, respectively. This difference is expected, since all of the aforementioned lesions are very common lesions in oral cavity. Another study has demonstrated hyperkeratosis to be the most frequent diagnosis (10.04%), followed by dentigerous cyst (6.98%) [20]. In our study, dentigerous cyst accounts for 4.7% of cases. In the present study, lichen planus appears to be the second most common diagnosis encountered (10.8%). Also, other studies showed it to be the third most common diagnosis in both Saudi Arabia and Spain, 7.1%, 14.1%, respectively [18, 25]. However, it was reported as the sixth most common lesion in a different study conducted in Kuwait (4.56%) [20]. This difference can also be related to the protocol of biopsy of such lesions as well. Many oral lichen planus can be diagnosed based on their clinical presentation without biopsy, particularly classic presentation [26–28]. Ameloblastoma was the most common odontogenic tumor, followed by odontoma. Fluctuant frequency of both lesions was noted in the literature according to countries. These disparities could be attributed not only to ethnicity, but also to discrepancies in survey methodology and sample sizes. Also, the prevalence of odontoma may be underestimated because of uncommon related symptoms as well as not being always considered for biopsy [29]. Regarding salivary glands pathology, the most common diagnosis was Sjogren's syndrome (3.15%). In contrast, a previous study has showed mucocele (4.3%) to be the most prevalent salivary gland pathology, followed by Sjogren's syndrome (0.5%) [25]. Mucocele is one of the most common salivary gland lesions. However, in this study, it came after Sjogren's syndrome. This also might be attributed to the awareness of people to get biopsy of such trauma-related lesion [30]. In regards to malignancies, the most common malignant tumor found in our study was squamous cell carcinoma (4.7%), which is in accordance with the findings of multiple previous studies [18, 24, 25, 31].

The most common lesion in females was lichen planus. The exact cause is unknown, however, literature suggests that they may be triggered by endogenous factors such as stress or anxiety, which appears to be more common in females [32]. This finding is consistent with previous studies [20, 32]. On the other hand, radicular cysts occur in males more than females. Radicular cysts are primarily consequent of pulp necrosis. The possible explanation of higher frequency of radicular cysts between males in this study is that males are prone to neglect oral hygiene, dental treatment, and follow-ups in absence of symptoms compared to female patients [33, 34].

One of the limitations of the current study is the insufficient detailed documentation of some cases. In addition, some demographic data associated with diagnoses such as socioeconomic status, oral habits, and occupation were not mentioned in the reports, such information might help establish more knowledge on these lesions and their risk factors. Also, the findings are based on general pathologists' diagnoses only without being reviewed by oral pathologists to confirm such lesions in the OMF area. De Andrade Melo et al. highlighted the importance of oral pathologists in the diagnosis of oral and maxillofacial lesions as they are familiar with the overlapping features of lesions in the area. This prevents avoidable misdiagnosis of such lesions [35]. Further studies are needed on oral and maxillofacial lesions diagnosed and documented by oral pathologists to ensure more accurate representation of such lesions.

### 4.1. Recommendation

The authors suggest studies revising the histopathologic slides of the lesions rather than relying on the reports only. Moreover, engaging oral pathologists in hospitals to participate in the diagnosis of OMF lesions are recommended to make more accurate diagnosis of these lesions.

## 5. Conclusion

In conclusion, the findings provide important information about the oral and maxillofacial pathology in Al-Qassim region, Saudi Arabia. This study found that biopsied oral lesions were more prevalent in males and in patients in the fourth decade of life. The oral mucosa NOS was the most biopsied site and the majority of the biopsies were soft tissue pathological lesions and radicular cyst was the most frequent diagnosis. Knowledge of such demographic and clinical features of oral and maxillofacial pathology cases helps in prediction of disease incidence and hence proper patient care in the region.

## Figures and Tables

**Table 1:** Distribution of lesions main diagnosis according to the categories.

Category	Diagnosis	Frequency (*N*)	Percentage (%)	Location
Pulp and periapical lesion	Periapical granuloma	8	2.1%	Oral mucosa NOS, maxilla, mandible
	Periapical abscess	1	0.3%	Mandible

Odontogenic cyst	Inflammatory odontogenic cyst	—	—	—
	Radicular cyst	52	13.6%	Maxilla, mandible, palate
	Residual cyst	3	0.8%	Maxilla, mandible
	Developmental odontogenic cyst	—	—	—
	Dentigerous cyst	18	4.7%	Maxilla, mandible
	Calcifying odontogenic cyst	2	0.5%	Maxilla, mandible
	Odontogenic keratocyte	4	1.0%	Mandible
	Miscellaneous NS	5	1.3%	—

Nonodontogenic cyst	Nasopalatine cyst	7	1.8%	Maxilla
	Miscellaneous NS	—	—	—
	Neoplastic simple cyst	1	0.3%	Palate
	Epidermoid cyst	1	0.3%	Mandible
	Dermoid cyst	2	0.5%	Oral mucosa NOS, maxilla

Odontogenic tumor	Ameloblastoma	7	1.8%	Mandible
	Odontoma	2	0.5%	Maxilla, mandible
	Fibromyxoma	1	0.3%	Mandible
	Adenomatoid AOT	1	0.3%	Maxilla
	Myxoma	1	0.3%	Mandible

Salivary gland pathology	Inflammatory and obstructive lesion	—	—	—
	Sialadenitis	6	1.6%	Lip
	Sialolithiasis	2	0.5%	NA
	Salivary gland cyst	—	—	—
	Mucocele	6	1.6%	Lip
	Ranula	1	0.3%	NA
	Salivary gland benign tumor	—	0.0%	—
	Oncocytic papillary cystadenoma	1	0.3%	Oral mucosa NOS
	Cellular pleomorphic adenoma	5	1.3%	Maxilla, mandible, palate
	Salivary gland malignant tumor	—	—	—
	Mucinous adenocarcinoma	1	0.3%	Mandible
	Acinic cell carcinoma	1	0.3%	Oral mucosa NOS
	Mucoepidermoid carcinoma	1	0.3%	NA
	Salivary gland syndrome related	—	—	—
	Sjogren's syndrome	12	3.1%	Lip
	Salivary gland idiopathic	3	0.8%	Oral mucosa

Soft tissue pathology lesion	Soft tissue inflammation	1	0.3%	Maxilla
	Reactive soft tissue	—	—	—
	Focal fibrous hyperplasia	36	9.4%	Maxilla, mandible, palate, tongue, lip
	Pyogenic granuloma	31	8.1%	Oral mucosa NOS, mandible, Palate, tongue, lip
	Peripheral giant cell granuloma	2	0.5%	Mandible
	Peripheral ossifying fibroma	1	0.3%	Mandible
	Fibrous epulis	3	0.8%	Oral mucosa NOS, maxilla
	Soft tissue benign tumor	—	—	—
	Plexiform Schwannoma	2	0.5%	Oral mucosa NOS, tongue
	Lipoma	1	0.3%	Mandible
	Neurofibroma	1	0.3%	Oral mucosa NOS
	Soft tissue malignant tumor	—	—	—
	Rhabdomyosarcoma	2	0.5%	Mandible
	Soft tissue NS	—	—	—
	Acute on chronic inflammation	1	0.3%	Maxilla
	Fibrosis	1	0.3%	Lip
	Hematopoietic tumors	—	—	—
	Lymphoma	1	0.3%	Mandible
	Lymphoid hyperplasia	2	0.5%	NA
	Soft tissue tumor vascular lesion	—	—	—
	Hemangioma	12	3.1%	Oral mucosa NOS, mandible, palate, tongue, lip
	Angiokeratoma	1	0.3%	Oral mucosa

Bone pathology lesion	Bone infection/inflammation and necrosis	—	0.0%	—
	Osteomyelitis-foci of necrotic bone spicules	7	1.8%	Oral mucosa NOS, mandible, maxilla
	Osteonecrosis	1	0.3%	Maxilla
	ORN	1	0.3%	Oral mucosa, mandible
	Bone tumor/tumor-like lesions	—	—	—
	Central ossifying fibroma	6	1.6%	Oral mucosa NOS, mandible
	Fibrous dysplasia	2	0.5%	Mandible
	Central giant cells lesion	1	0.3%	Maxilla
	Desmoplastic fibroblastoma	1	0.3%	Maxilla
	Miscellaneous	—	—	—
	Langerhans's cell histiocytosis	1	0.3%	Mandible

Immunological-mediated lesions	Lichen planus	41	10.8%	Oral mucosa NOS, mandible
	*Pemphigus vulgaris*	4	1.0%	Oral mucosa NOS, lip
	Aphthous ulcer	1	0.3%	Tongue
	Erythema multiform	1	0.3%	Oral mucosa NOS
	Lichenoid reaction	4	1.0%	Oral mucosa NOS, tongue

Epithelial lesion	Frictional hyperkeratosis	1	0.3%	Oral mucosa NOS
	Hyperkeratosis lesion	5	1.3%	Oral mucosa NOS, tongue
	Epithelial benign tumor-HPV-related lesions	—	—	—
	Focal epithelia hyperplasia (hecks disease) HPV virus	1	0.3%	NA
	Wart	1	0.3%	Lip, palate
	Squamous papilloma	8	2.1%	Tongue, lip

Premalignant	Focal atypia	2	0.5%	Maxilla, tongue
	Leukoplakia	3	0.8%	Oral mucosa NOS
	Cheilitis	1	0.3%	Oral mucosa NOS
	Epithelial malignant tumor	—	—	—
	Squamous cell carcinoma	18	4.7%	Oral mucosa NOS, mandible, tongue, lip
	Melanoma	1	0.3%	Palate
	Atypical squamoproliferative lesion	1	0.3%	Maxilla
	Adenosquamous carcinoma	1	0.3%	Tongue
	Adenocarcinoma	3	0.8%	Mandible

Fungal infection	Mucormycosis	2	0.5%	Palate, maxillary sinus

Gingival-related lesion	Gingival infection/inflammation	—	—	—
	Plasma cell gingivitis	3	0.8%	Oral mucosa NOS
	Gingival reactive lesion	—	0.0%	—
	Drug-related gingival hyperplasia	1	0.3%	Maxilla
	Gingival-related lesion-miscellaneous NS	—	—	—
	Fibromatosis	3	0.8%	Oral mucosa NOS, palate

Ulcerative	Mucosal nonspecific ulcer	1	0.3%	Mandible
	Bleeding nonspecific ulcers	1	0.3%	NA

	Total	381	100.0%	—

## Data Availability

We would like to confirm that the data that support the findings of our study are available upon request from the corresponding author. The data are not publicly available to ensure privacy protection of research participants.
